# Moroccan entomopathogenic nematodes as potential biocontrol agents against *Dactylopius opuntiae* (Hemiptera: Dactylopiidae)

**DOI:** 10.1038/s41598-022-11709-4

**Published:** 2022-05-09

**Authors:** Mohamed El Aalaoui, Fouad Mokrini, Abdelfattah A. Dababat, Rachid Lahlali, Mohamed Sbaghi

**Affiliations:** 1 Plant Protection Department, National Institute of Agricultural Research, Ennasr Rabat, BP 415 RP, Rabat, Morocco; 2 Biotechnology Research Unit, National Institute of Agricultural Research (INRA), CRRA, 10000 Rabat, Morocco; 3 International Maize and Wheat Improvement Center (CIMMYT), Emek, Ankara, 06810 Turkey; 4 Phytopathology Unit, Department of Plant Protection, Ecole Nationale d’Agriculture de Meknès, Km10, Rte Haj Kaddour, BP S/40, 50001 Meknès, Morocco

**Keywords:** Ecology, Plant sciences

## Abstract

*Dactylopius opuntiae* (Cockerell) (Hemiptera: Dactylopiidae) or prickly pear cochineal, is the most damaging pest on cactus species with heavy economic losses worldwide. The efficacy of two Moroccan EPN isolates; *Steinernema feltiae* (Filipjev) (Rhabditida: Steinernematidae) and *Heterorhabditis bacteriophora* (Poinar) (Rhabditida: Heterorhabditidae) (applied at 25, 50, and 75 IJs cm^−2^) against *D. opuntiae* nymphs and young females were evaluated under both laboratory bioassays and field conditions. Results showed that *S. feltiae* was more effective, causing higher mortality of nymphs and adult females (98.8% and 97.5%, respectively) after 8 days of exposure, resulting in an LT_50_ value of 5.9 days (nymph) and 6.0 days (young female). While, *H. bacteriophora* had lower mortalities (83.8% for nymph and 81.3% for adult females). For the cochineal nymphs and adult females, no significant difference was observed among *S. feltiae* at 25, 50, and 75 IJs cm^−2^, and the positive control, d-limonene applied at 0.5 g/L which was used due to its high effectiveness against nymphs and females of *D. opuntiae*. In the field experiment, d-limonene at 0.5 g/L and *S. feltiae* applied at 75 IJs cm^−2^ were effective in reducing nymph and adult female populations by 85.3–93.9% at 12 days of post exposure period. To our knowledge, this work is the first report on the use of EPNs to control *D. opuntiae*. Thus, in addition to d-limonene, both Moroccan EPN isolates *S. feltiae*, and *H. bacteriophora* could be used as part of the integrated pest management strategy against *D. opuntiae*. Many factors such as temperature can affect the establishment and effectiveness of EPNs under field conditions. Therefore, additional studies under field conditions are needed.

## Introduction

Native to Mexico, the cactus cochineal *Dactylopius opuntiae* (Cockerell) (Hemiptera: Dactylopiidae), is an invasive pest of prickly pear, *Opuntia ficus-indica* (L.) Mill. (Caryophyllales: Cactaceae) and other Opuntia species in the Americas, Asia, Europe, and Africa^1,2^. In 2014, *D. opuntiae* was detected for the first time in Morocco^3^. Both nymphs and female adults stages of *D. opundiae* feed directly and permanently on the host plant's elaborate sap causing chlorosis and premature dropping of cladodes and fruits^4^. Severe infestations of up to 50% of the cladode surface can in some time lead to the death of the plant^5,6^. In Brazil, the damage caused by *D. opuntiae* on *O. ficus indica* and other Opuntia species used as forage resulted in the loss of 100,000 ha, valued at $25 US million^7^. Similarly, in Mexico, the damage caused by this pest is severe, the premature drop of fruits and young cladodes has resulted in lower yields and higher production costs for Mexican cactus crops^8^. Since its first detection in Morocco, the introduced *D. opuntiae* has caused enormous damage in several areas of cactus production in the country where prickly pear cactus plays an essential role in the ecological system, preventing desertification and preserving biodiversity^9^. *Dactylopius opuntiae* establish and spread more easily than many other scale insect species due to the waxy coating on their dorsal side which protects them from exposition to contact insecticides, high reproductive rate, and the propensity to spread quickly through natural carriers such as plant products, wind, water, rain, birds, human, and farm animals^10^. *Dactylopius opuntiae* females have three biological developmental stages—egg, nymph (two instars), and adult—whereas males have egg, nymph (1st, 2nd, 3rd, 4th, and 5th), pre-pupa, pupa, and adult^11^. For male the moults to the third, fourth and fifth (nymph) instars take place in the cocoon, but the successive exuviae are extruded from the posterior end of the cocoon, indicating when the moult has occurred^11^. *Datylopius opuntiae* has high fecundity rate (on average 150–160 eggs) with total incubation period < 1 day^8^. The average developmental time for *D. opuntiae* crawler from egg to first moult is 14.92 days, the mean duration of the female from egg to the commencement of oviposition is 84 days^11^. The duration of the second instar from the first moult to the commencement of pupation averaged 11.2 days^10^. The pupal period lasted approximately 10 days, at the end of which emerged a red, white-winged male^11,12^. The adult male is short lived, usually dies within 3–5 days after its emergence and does not consume during this period of life^11^. The life cycle of *D. opuntiae* varies from 90 to 136 days depending on the environmental conditions, especially the temperature (the cycle duration is short in high temperatures)^12^. They also seem to be able to become dormant on inert material over the period of time under unfavorable conditions^11^.

*Dactylopius opuntiae* is controlled mainly by organophosphate and neonicotinoids insecticides^8^, which can have harmful effects on human and animal health^13^, the environment^14^, and in some cases limit international trade in cactus plants among the countries interested in cactus cultivation, including Morocco^4^. Therefore, to reduce insecticide use, many alternative management strategies have been explored in many countries, such as the use of mineral oils, resistant genotypes, detergents, plant extracts, mycoinsecticides, and biological control agents (Predators)^4,15,16^.

The *Fusarium incarnatum*-equiseti species complex alone has been used in Brazil to control *D. opuntiae*^17^ or in combination with natural extracts of *Ricinus communis* L. and *Poincianella pyramidalis* (Tul.) L.P. Queiroz, and results are promising^18^. A key point for the use of these fungi in IPM is the molecular characterization of the genetic profiles of *F. incarnatum*-equiseti strains, as there are considerable differences in efficacy as biological control agents among the biotypes collected in the field^17,19^. Several species of predators (insects and spiders) are associated with *D. opuntiae* and some can control it, but to date, no parasitoids have been found associated with *D. opuntiae*. To prevent the spread of the *D. opuntiae* in Morocco, the Ministry of Agriculture, Maritime Fisheries, Rural Development and Water and Forests, implemented a major emergency plan for the control of this scale pest in 2016. This plan also included a research program covering the most important management components such as biopesticides^20^, beneficial insects^21–23^, and host plant resistance^9^. Five botanical extracts and one detergent were tested for the control of nymphs and adult females of *D. opuntiae* in laboratory bioassays and under field conditions in Morocco. The results show that the use of biodegradable products, black soap at 60 g/l in double application or in combination with *Capsicum annuum* L. extract at 200 g/l, could be incorporated into the management program for the control of *D. opuntiae* as a safe alternative to chemical insecticides^20^. Also, after the detection of *D. opuntiae* in Morocco, investigations were carried out in different areas of cactus production in order to find biological control agents that could be used as predators against *D. opuntiae*. Fourteen predators were found associated with *D. opuntiae* and identified^22^. *Hyperaspis campestris* (Herbst, 1783) was found to be the most important species associated with *D. opuntiae* in Morocco^22^. It is important to note that the varietal resistance of cactus to *D. opuntiae*, was the relevant and saving solution currently exists in Morocco. The eight cactus varieties identified as resistant to *D. opuntiae* by research in Morocco, will constitute a solid base for the launching of the national program of recovery of decimated cactus throughout the country^9^. Since the body of *D. opuntiae* is covered by wax that makes it hard to be controlled by the use of chemical and botanical sprays, therefore, biological control seems to be promising alternative control strategies^24^ than insecticides. Within biological control, entomopathogenic nematodes (EPNs) of the families Heterorhabditidae and Steinernematidae are obligate pathogens of insects^25^, associated with specific symbiotic bacteria belonging to the genera *Photorhabdus* and *Xenorhabdus* respectively^26^. The life cycle of EPN consists of four stages, egg, juvenile (four stages), adult, and the 3^rd^ juvenile infective stage^27^. Regarding the mode of action of EPNs, the infective juveniles enter the host body and release their bacteria, and subsequently the insect dies within 48 h after infection^28,29^. EPNs complete 1–3 generations in a single host and when the necessary resources are depleted, the IJs leave the host and forage for a new situable host^27^. The developmental duration and reproduction of EPNs depends on host quality, however, information on their host searching behavior under field conditions is quite scarce in the literature^30^. The host-seeking behavior of EPNs has been classified into two categories: cruisers (active seekers) and ambushers (sit-and-wait foragers)^31,32^. Crusaders, such as *H. bacteriophora*, are highly mobile and active^31,32^, with an ability to orient themselves based on long-range volatile signals^33,34^ and an ability to find sedentary subterranean hosts^34^. On the other hand, ambushers, such as *Steinernema carpocapsae* Weiser (Rhabditida: Steinernematidae), have low mobility^31,32^, an ability to nest or stand^32^, and a lack of response to long-range volatile signals^33,34^. But some species, such as *S. feltiae*, do not nest like ambushers^32^ and do not respond to long-range volatile host signals in a manner similar to cruisers^34^. EPNs are very promising biocontrol agents showing high potential for the management of many harmful pests of different crops including scale pests^35–45^. Several strains of *Steinernema feltiae* and *Heterorhabditus bacteriophora* native to Morocco have been identified^46–48^. To the best of our knowledge, the efficacy of these native EPN isolates, isolated from Moroccan soils, has not been evaluated against *D. opuntiae*. Therefore, in this study, we evaluated the effectiveness of two indigenous EPNs in controlling nymphs and adult females of *D. opuntiae* under laboratory and field conditions.

## Results

### Laboratory trials

**Figure 1:**
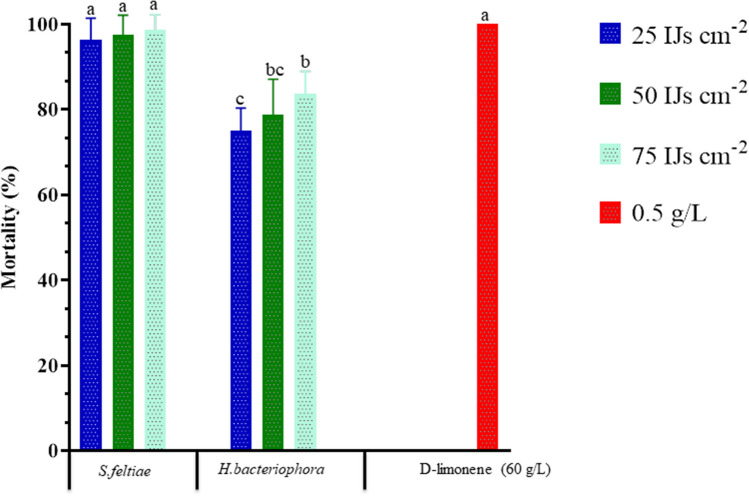
Comparative efficiency of two entomopathogenic nematodes isolates *Steinernema feltiae* and *Heterorhabditis bacteriophora* with d-limonene against *Dactylopius opuntiae* nymphs, in laboratory assay. Datasets are the mean of two independent trials with four relicates. Treatments with the same letter are not significantly different according to Tukey's LSD test at P < 0.05.

**Figure 2:**
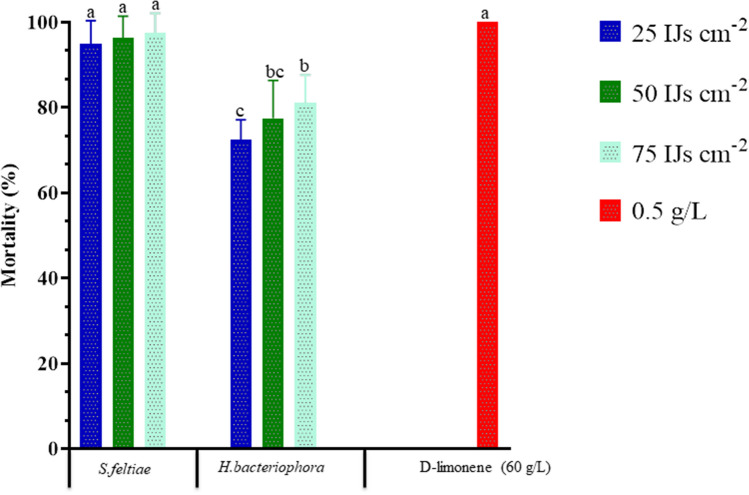
Comparative efficiency of different entomopathogenic nematodes isolates *Steinernema feltiae* and *Heterorhabditis bacteriophora* with d-limonene against *Dactylopius opuntiae* young females, in laboratory assay. Datasets are the mean of two independent trials with four relicates. Treatments with the same letter are not significantly different according to Tukey's LSD test at P < 0.05.

The Moroccan EPN isolates *S. feltiae* and *H. bacteriophora* were evaluated for their pathogenicity against *D. opuntiae* nymphs and adult females based on their infectivity rates. The tested EPN species at different concentrations were significantly different in their pathogenicity against nymphs (Fig. 1) and adults female of *D. opuntiae* (Fig. 2). The greatest 8-days nymph cumulative mortality (96.3–100%) was achieved by d-limonene applied at 0.5 g/L, and *S. feltiae* at 25, 50, and 75 IJs cm^−2^ (*F*_*index*_ = 33.30, df = 6; 56, *P* < 0.0001). At the end of the experiment, nymph cumulative mortality for *H. bacteriophora* at high concentration (75 IJs cm^−2^) reached 83.8%.

**Figure 3:**
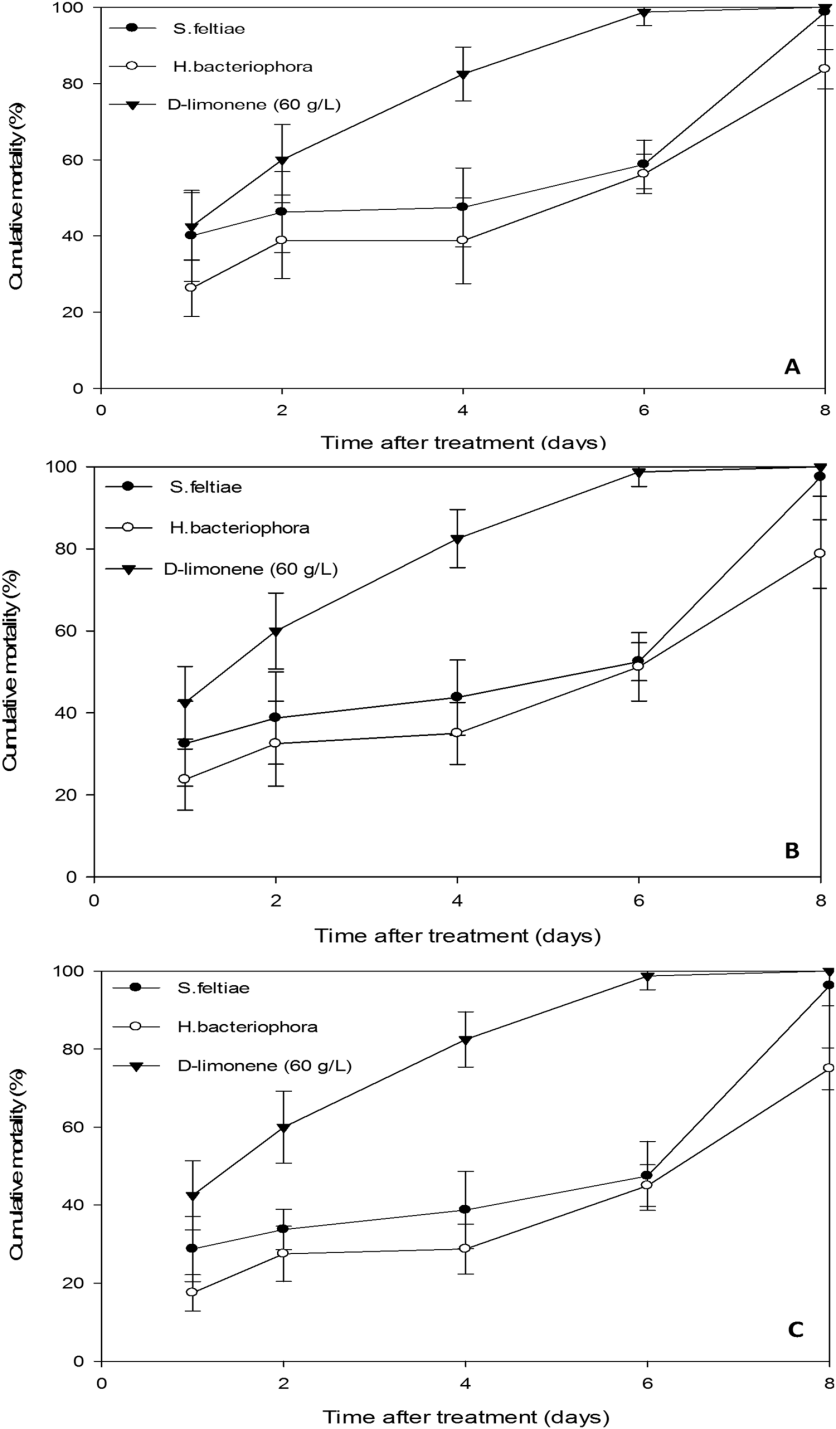
Cumulative mortality of *Dactylopius opuntiae* nymphs by *Steinernema feltiae* and *Heterorhabditis bacteriophora* with different concentrations (**A**—75, **B**—50, **C**—25 IJs cm^−2^) according to days after treatment. Vertical bars indicate standard error.

At 8 days after treatment, the greatest adult mortality was achieved by d-limonene at 0.5 g/L (100%), and *S. feltiae* at 25, 50, and 75 IJs cm^−2^ (95.0–97.5%) (*F*_*index*_ = 33.3, df = 6; 56, *P* < 0.0001). (*F*_*index*_ = 31.9, df = 6; 56, *P* < 0.0001). There was no significant difference in the percentage mortality caused by *H. bacteriophora* at 50 and 75 IJs cm^−2^. The lowest percentage of adult female mortality observed at 8 days after treatment was achieved by *H. bacteriophora* at 25 IJs cm^−2^ (72.5%). The pathogenic efficacy of all nematodes tested against *D. opuntiae* nymph increased as the exposure period increased. The largest increase, from day 1 to day 8 post-treatment, was seen between d-limonene and *S. feltiae* at the concentration of 75 IJs cm^−2^. The experimental data are presented in Fig. 3.

During the first day after treatment, *D. opuntiae* nymph mortality at the highest concentration showed a 40% variant for both d-limonene and *S. feltiae*. The same mortality rates (40%) were displayed in *H. bacteriophora*, but at day 4 after treatment. Maximum mortality was recorded after 8 days by both d-limonene (100%) and *S. feltiae* (98.8%) (Fig. 3A).

For the concentration of 50 IJs cm^−2^. At 24 h post-treatment, the highest nymphal mortality was achieved by d-limonene (42.5%) followed by *S. feltiae* (32.5%), whereas 2 days are required for *H. bacteriophora* to kill the same number of insects (32.5%). After 8 days post-treatment, d-limonene and *S. feltiae* reached 100% and 97.5% mortality, respectively, while *H. bacteriophora* reached 78.8% mortality (Fig. 3B).

The lowest percentage of nymphal mortality was observed at the concentration of 25 IJs cm^−2^ (Fig. 3C). At 24 h post-treatment, the highest nymphal mortality was achieved by *S. feltiae* (28.8%), whereas 4 days are required for *H. bacteriophora* to kill the same number of scale insects (28.8%). After 8 days post-treatment, d-limonene and *S. feltiae* reached 100% and 96.3% mortality, respectively, while *H. bacteriophora* reached 75% mortality (Fig. 3C).

**Figure 4:**
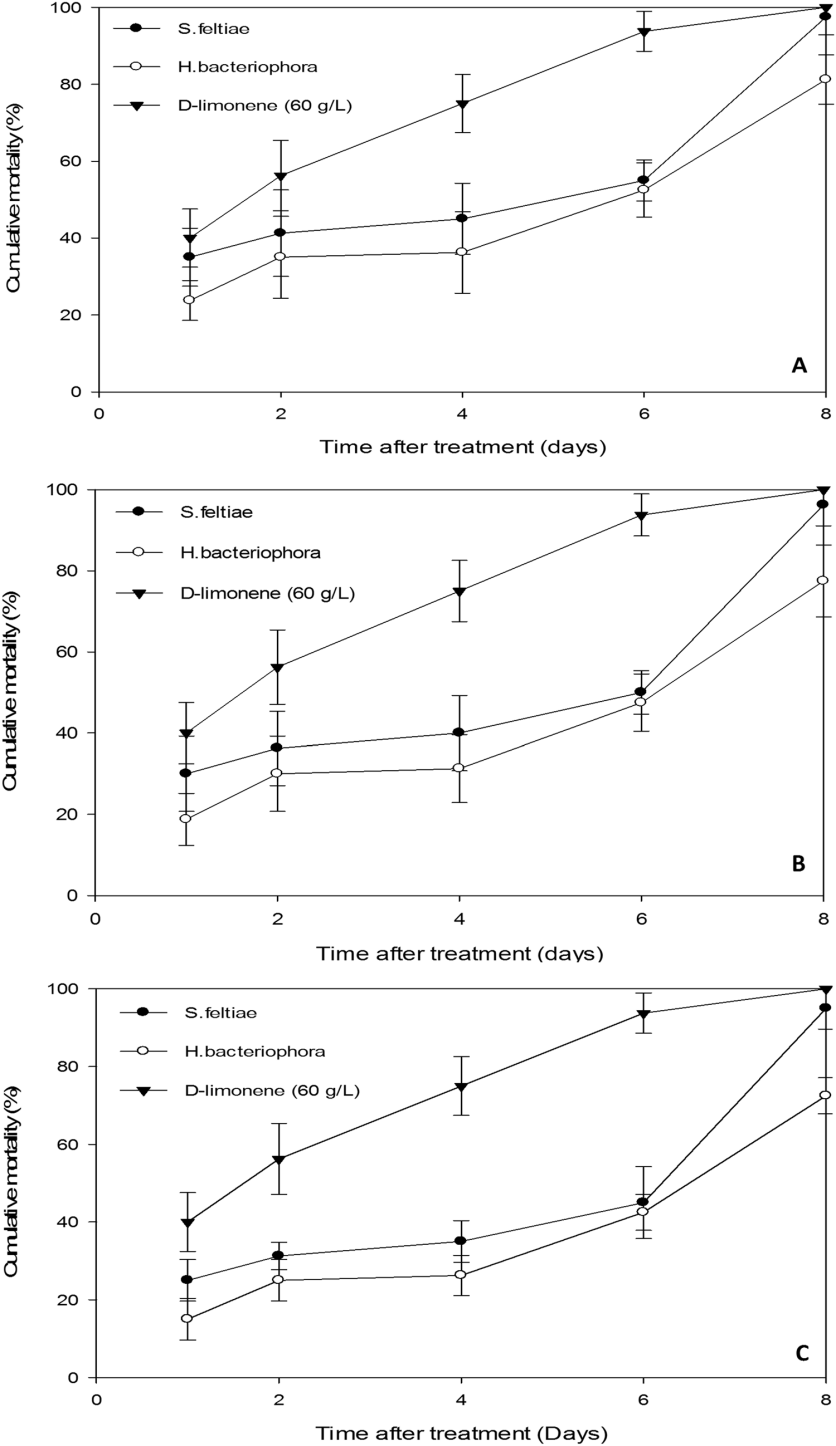
Cumulative mortality of *Dactylopius opuntiae* adult’s female by *Steinernema feltiae* and *Heterorhabditis bacteriophora* with different concentrations (**A**—75, **B**—50, **C**—25 IJs cm^−2^) according to days after treatment. Vertical bars indicate standard error.

**Figure 5:**
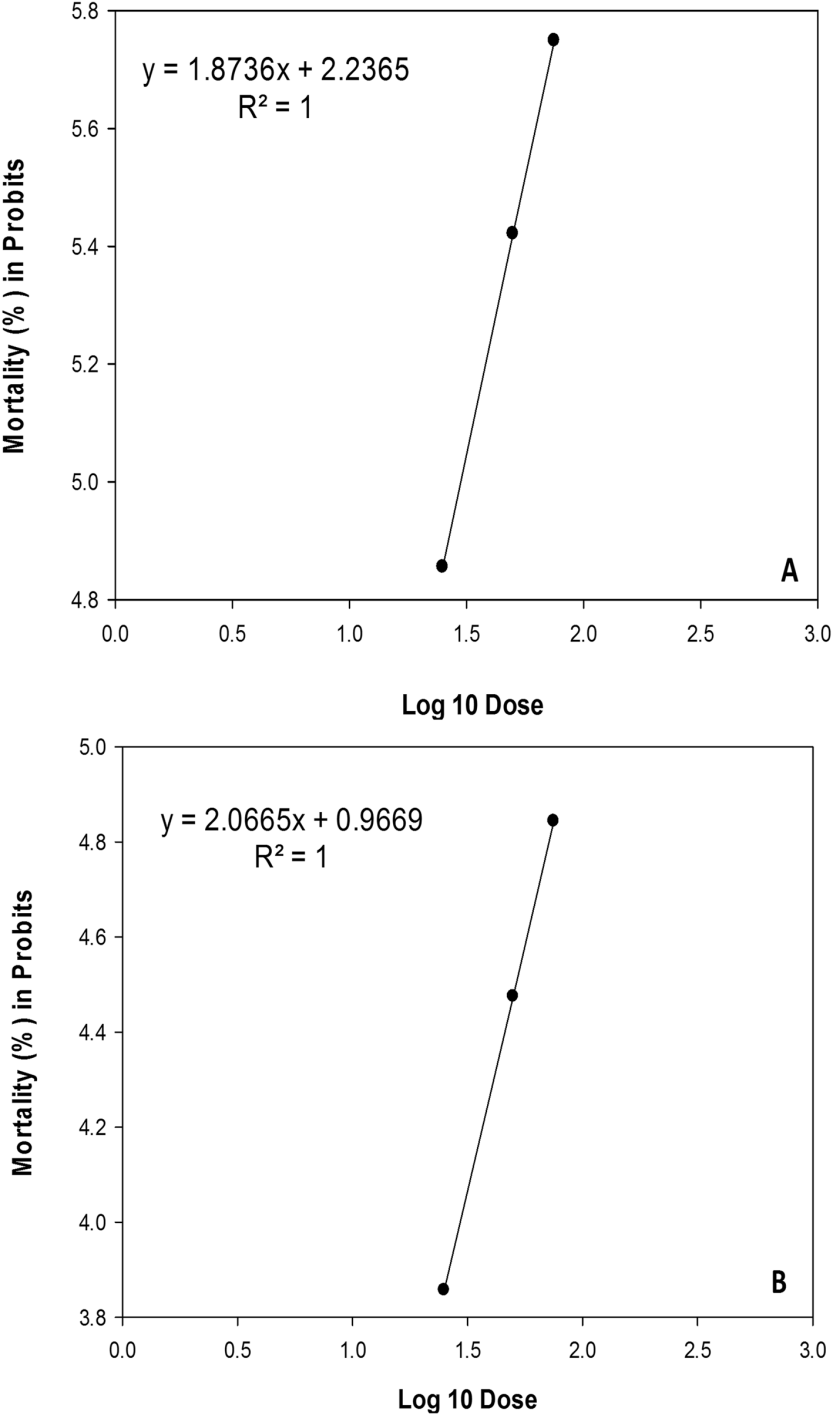
Probit mortality of *Dactylopius opuntiae* nymphs exposed to the two EPNs different concentrations: (**A**) *Steinernema feltiae*, (**B**) *Heterorhabditis bacteriophora*.

A significant difference in nematodes pathogenicity against adult’s female was also observed among treatments (Fig. 4). At the highest concentration (75 IJs cm^−2^), the highest mortality percentages at 24 h were observed among adult’s female exposed to d-limonene (40%) and *S. feltiae* (35%), whereas 2 days are required for *H. bacteriophora* to kill the same number of scale insects (35%). *Steinernema feltiae* and *H. bacteriophora* reached mortality of 97.5% and 81.3%, respectively, at the 8 th day post-infection (Fig. 4A). At the concentration of 50 IJs cm^−2^, mortality was achieved 30% on day 3 and 96.3% on day 8 by *S. feltiae*. Two days are required for *H. bacteriophora* to kill the same number of insects (30%). After 8 days post-treatment, *H. bacteriophora* reached 77.5% mortality of *D. opuntiae* adult females (Fig. 5B). Low mortality was observed at the concentration of 25 IJs cm^−2^ (Fig. 4C). With the exception of *S. feltiae*, which killed 25% and 95% of the insects tested at days 1 and 8 post-treatment, respectively; *H. bacteriophora* achieved 15% mortality at day 1 and 26.3%, 42.5%, 72.5%, respectively, at days 4, 6, and 8 post-infections.

The susceptibility of *D. opuntiae* to a particular nematode species and concentration is one of the major factors determining LC_50_ levels. The concentrations required to induce 50% mortality of *D. opuntiae* nymphs and young females under the effect of *S. feltiae* and *H. bacteriophora* are shown in Table 1. The Probit analysis used to analyze the mortality results showed that *S. feltiae* had the lowest median lethal concentration value, whereas *H. bacteriophora* had the highest (Table 1).

**Table 1:** Median lethal concentration LC_50_ (IJs cm^−2^) of *Dactylopius opuntiae* treated by *S. feltiae* and *H. bacteriophora* (ANOVA, α = 0.05).

Treatments	The *D. opuntiae* stage	Slope ± SE	LC_50_ (IJs cm^−2^)	Chi-test (χ^2^) Sig	df	P-value
*S. feltiae*	Nymph	0.47 ± 0.18	29.84	286.15 (P < 0.0001)	118	0.010
	Adult female	0.44 ± 0.18	42.52	296.44 (P < 0.0001)	118	0.017
*H. bacteriophora*	Nymph	0.52 ± 0.19	89	217.78 (P < 0.0001)	118	0.004
	Adult female	0.50 ± 0.19	129.28	226.67 (P < 0.0001)	118	0.007

**Figure 6:**
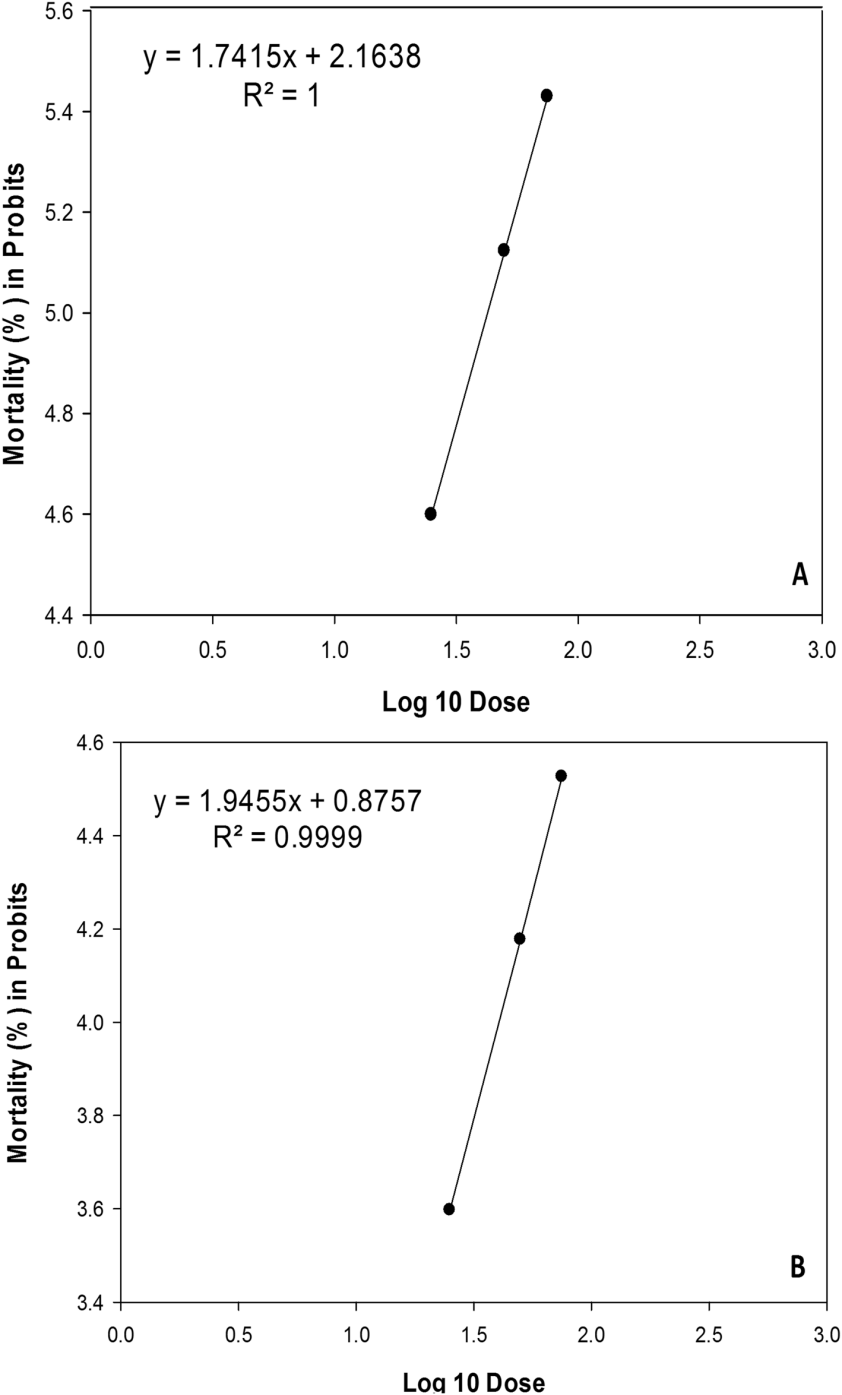
Probit mortality of *Dactylopius opuntiae* adult’s female exposed to the two EPNs different concentration: (**A**) *Steinernema feltiae*, (**B**) *Heterorhabditis bacteriophora*.

The concentration required to induce 50% mortality of *D. opuntiae* nymphs and young females for the two EPN isolates tested at low, medium, and high concentrations are shown in Fig. 5A,B (nymph) and Fig. 6A,B (young female). One-way ANOVA analysis shows that mean mortality was significantly (P ≤ 0.05) affected by exposure of *D. opuntiae* insects to different concentrations of EPN suspensions. Insects exposed during the period from 1 to 8 days after treatment to the highest concentration (75 IJs cm^−2^), exhibited a significantly higher mortality rate.

**Figure 7:**
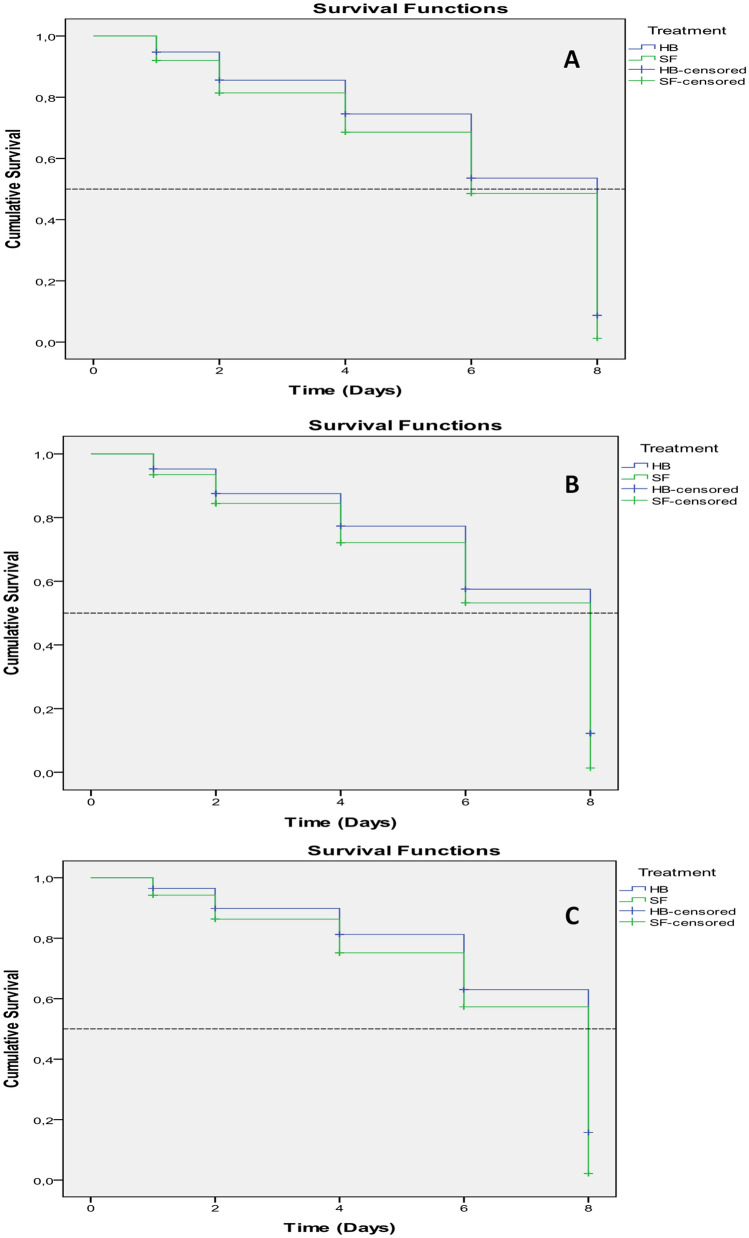
Survival curves (Lethal time LD_50_) of *Dactylopius opuntiae* nymphs treated by different concentrations of the two EPNs: (**A**) 75 IJs cm^−2^, (**B**) 50 IJs cm^−2^, (**C**) 25 IJs cm^−2^.

**Figure 8:**
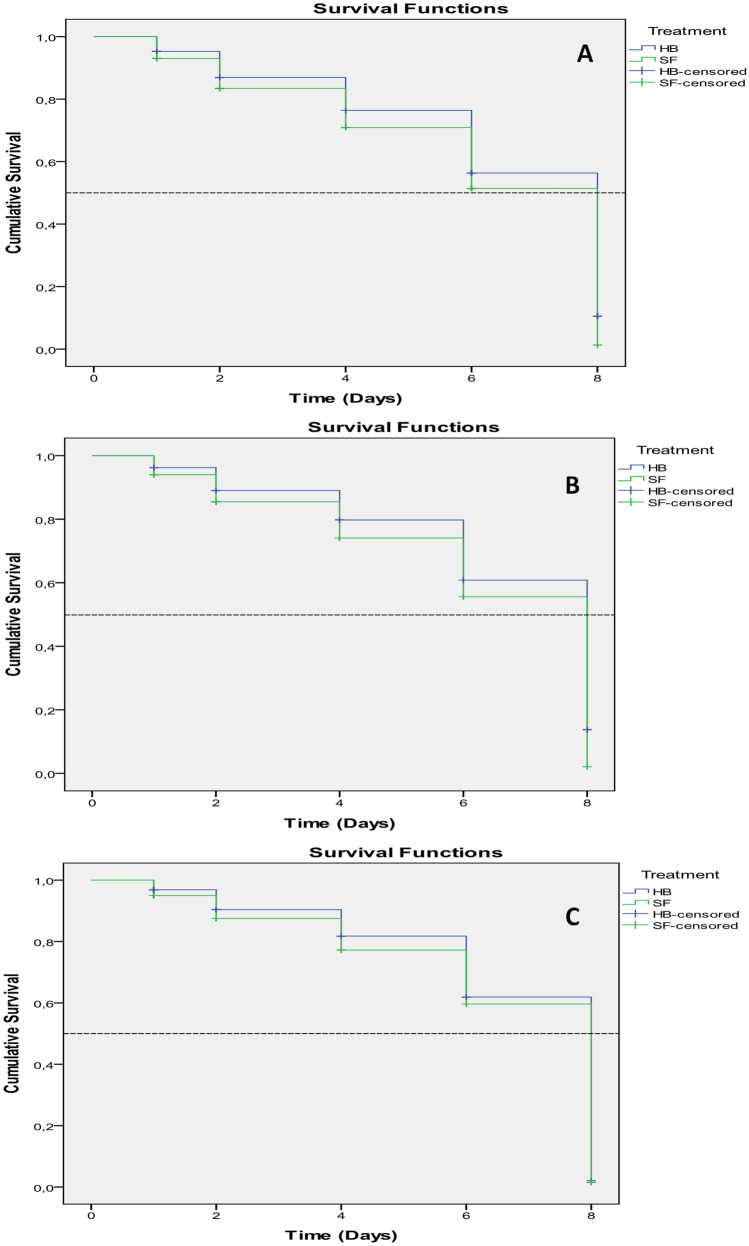
Survival curves (Lethal time LD_50_) of *Dactylopius opuntiae* adults female treated by different concentrations of the two EPNs: (**A**) 75 IJs cm^−2^, (**B**) 50 IJs cm^−2^, (**C**) 25 IJs cm^−2^.

The mean survival time (LT_50_) of *D. opuntiae* nymphs (Fig. 7A) and young females (Fig. 8A) exposed to selected nematodes with a concentration of 75 IJs cm^−2^ ranged from a minimum of 5.9 to a maximum of 6.2 days and from a minimum of 6.2 to a maximum of 6.4 days, respectively (Table 2). The survival curves for all treatments were different by the Kaplan–Meier method (P < 0.05). Pearson's chi-square statistical test (all P values < 0.05) indicated that the data did not fit the regression models according to Breslow (generalized Wilcoxon), where χ^2^ = 4.25, df = 1, sig = 0.039 (nymph) and χ^2^ = 3.86, df = 1, sig = 0.05 (young female).

**Table 2:** Mortality (%), mean survival time, and LT_50_ (days) of *Dactylopius opuntiae*-treated nematode isolates with concentration 75 IJs cm^−2^.

Treatments	The *D. opuntiae* stage	Mortality (%)^a^	Mean survival time ± SE^b^	LT_50_ (95% CI) days	N^c^
*S. feltiae*	Nymph	58.1	5.9 ± 0.1	6.0	80
	Adult female	54.7	6.0 ± 0.1	8.0	80
*H.bacteriophora*	Nymph	48.7	6.2 ± 0.1	8.0	80
	Adult female	45.6	6.4 ± 0.1	8.0	80

^a^Abbott-corrected percentage mortality of *D. opuntiae* nymphs and adults female at the end of experiment.

^b^The mean survival time and its standard error.

^c^Total number of *D. opuntiae* in bioassay.

Lethal survival time analysis of *D. opuntiae* nymphs and young females exposed to the control and selected nematodes with concentration 50 IJs cm^−2^ did not indicate any significant difference between mortality times (Table 3). The survival curves for treatments with the 50 IJs cm^−2^ concentration were different by the Kaplan–Meier method (P < 0.05). Pearson's chi-square statistical test for nymphs (all P values > 0.05) (Fig. 7B) indicated that the data fit the regression models, where χ^2^ = 3.28, df = 1, sig = 0.07 and concerning young females (Fig. 8B) χ^2^ = 4.56, df = 1, sig = 0.033 according to Breslow (generalized Wilcoxon). Likewise, with the 25 IJs cm^−2^ concentration, no significant difference was observed between the mortality times (Table 4). As for the other concentrations tested, the survival curves for the treatments with 25 IJs cm^−2^ concentration were different by the Kaplan–Meier method (P < 0.05) but the Pearson chi-square statistical test (all P values > 0.05) indicated that the data fit the regression models for the young females (Fig. 8C), where χ^2^ = 1.92, df = 1, sig = 0.16 whereas for the nymphs (Fig. 7C) χ^2^ = 5.29, df = 1, sig = 0.021 according to Breslow (generalized Wilcoxon).

**Table 3:** Mortality (%), mean survival time, and LT_50_ (days) of *Dactylopius opuntiae*-treated nematode strains with concentration 50 IJs cm^−2^.

Treatments	The *D. opuntiae* stage	Mortality (%)^a^	Mean survival time ± SE^b^	LT_50_ (95% CI) days	N^c^
*S. feltiae*	Nymph	53.0	6.1 ± 0.1	8.0	80
	Adult female	50.5	6.2 ± 0.1	8.0	80
*H.bacteriophora*	Nymph	44.1	6.4 ± 0.1	8.0	80
	Adult female	41.0	6.7 ± 0.1	8.0	80

^a^Abbott-corrected percentage mortality of *D.opuntiae* nymphs and adults female at the end of experiment.

^b^The mean survival time and its standard error.

^c^Total number of *D. opuntiae* in bioassay.

**Table 4:** Mortality (%), mean survival time, and LT_50_ (days) of *Dactylopius opuntiae*-treated nematode isolates with concentration 25 IJs cm^−2^.

Treatment	The *D. opuntiae* stage	Mortality (%)^a^	Mean survival time ± SE^b^	LT_50_ (95% CI)	N^c^
*S. feltiae*	Nymph	49.0	6.3 ± 0.1	8.0	80
	Adult female	42.5	6.4 ± 0.1	8.0	80
*H.bacteriophora*	Nymph	38.7	6.7 ± 0.1	8.0	80
	Adult female	38.2	6.6 ± 0.1	8.0	80

^a^Abbott-corrected percentage mortality of *D. opuntiae* nymphs and adults female at the end of experiment.

^b^The mean survival time and its standard error.

^c^Total number of *D. opuntiae* in bioassay.

### Field trials

At 3 DAT, fewest nymphs were recorded in cactus plants treated with d-limonene at 0.5 g/L, and *S. feltiae* at 75 IJs cm^−2^ (*F*_*index*_ = 375.0, df = 7; 64, *P* < 0.0001) (Table 5). The numbers of nymphs in plants treated with d-limonene at 0.5 g/L, *S. feltiae* at 75 IJs cm^−2^, and *H. bacteriophora* at 75 IJs cm^−2^ were significantly reduced at 6 DAT (*F*_*index*_ = 796.5, df = 7; 64, *P* < 0.0001). At 12 DAT, nymphs density in control plants increased to 180 individuals but was significantly lower in all other treatments, with fewest scale pests in plants treated with d-limonene at 0.5 g/L, *S. feltiae* at 75 IJs cm^−2^ and *H. bacteriophora* at 75 IJs cm^−2^ (*F*_*index*_ = 3797.6, df = 7; 64, P < 0.0001). In general, d-limonene at 0.5 g/L, and *S. feltiae* at 75 IJs cm^−2^ were effective at reducing nymphs numbers, with over 90% reductions at 12 DAT when compared to the control (tap water) (Table 6) (*F*_*index*_ = 123.2, df = 6; 56, *P* < 0.0001).

**Table 5:** Average number of *Dactylopius opuntiae* nymphs alive at 1-day pre-treatment and 3, 6, and 12 days after been exposed to *S. feltiae*, *H. bacteriophora*, and d-limonene (60 g/l) (DAT), or tap water under field conditions.

Treatments	Concentration	Average number (± SE) at			
		1 d-Pre-trt	3 DAT	6 DAT	12 DAT
*S. feltiae*	25 IJs cm^−2^	150.0 ± 0.0	105.3 ± 5.5 b	78.1 ± 6.7 b	30.3 ± 4.3 b
	50 IJs cm^−2^	150.0 ± 0.0	82.4 ± 3.7 cd	52.8 ± 6.0 c	22.3 ± 3.4 c
	75 IJs cm^−2^	150.0 ± 0.0	71.3 ± 7.5 ef	40.5 ± 4.6 d	10.2 ± 2.0 e
*H. bacteriophora*	25 IJs cm^−2^	150.0 ± 0.0	110.2 ± 5.3 b	82.3 ± 6.7 b	34.0 ± 4.5 b
	50 IJs cm^−2^	150.0 ± 0.0	87.5 ± 3.7 c	57.3 ± 5.9 c	26.2 ± 3.1 c
	75 IJs cm^−2^	150.0 ± 0.0	76.1 ± 7.7 de	44.8 ± 6.1 d	14.3 ± 2.1 d
d-limonene (60 g/l)	0.5 g/L	150.0 ± 0.0	67.3 ± 8.1 f.	38.0 ± 4.5 d	7.7 ± 1.8 e
Control (tap water)	–	150.0 ± 0.0	169.7 ± 4.3 a	176.8 ± 3.6 a	180.4 ± 3.5 a

In each column, averaged means within followed by the same letters are not significantly different according to Tukey’s LSD test at α = 0.05.

**Table 6:** Henderson-Tilton adjusted rates of population reduction of *Dactylopius opuntiae* at 12 days after treatments under field conditions.

Treatments	Concentration	Rates of reduction (%) (mean ± SE) of	
		Nymph^a^	Adult^a^
*S. feltiae*	25 IJs cm^−2^	75.7 ± 3.4 d	69.2 ± 3.2 e
	50 IJs cm^−2^	82.2 ± 2.7 c	76.6 ± 1.9 c
	75 IJs cm^−2^	91.9 ± 1.6 a	85.3 ± 1.5 a
*H. bacteriophora*	25 IJs cm^−2^	72.7 ± 3.6 d	65.6 ± 3.2 f
	50 IJs cm^−2^	79.0 ± 2.5 c	73.4 ± 1.8 d
	75 IJs cm^−2^	88.6 ± 1.7 b	81.7 ± 1.4 b
d-limonene (60 g/l)	1.5 g/L	93.9 ± 1.4 a	86.6 ± 1.8 a

^a^In each column, averaged means followed by the same letters are not significantly different according to Tukey’s LSD test at α = 0.05.

d-limonene at 0.5 g/L, and *S. feltiae* at 75 IJs cm^−2^ significantly reduced young females densities in the treated cactus plants at 3 DAT (*F*_*index*_ = 319.2, df = 7; 64, *P* < 0.0001) (Table 7). Also, no significant difference was observed between *S. feltiae* 75 IJs cm^−2^ and *H. bacteriophora* 75 IJs cm^−2^ at 3 DAT.

**Table 7:** Average number of *Dactylopius opuntiae* young females alive at 1-day pre-treatment and 3, 6, and 12 days after been exposed to *S. feltiae*, *H. bacteriophora*, and d-limonene (60 g/l) (DAT), or tap water under field conditions.

Treatments	Concentration	Average number (± SE) at			
		1 d-Pre-trt	3 DAT	6 DAT	12 DAT
*S. feltiae*	25 IJs cm^−2^	150.0 ± 0.0	114.4 ± 4.1 b	87.0 ± 6.7 b	39.2 ± 4.1 c
	50 IJs cm^−2^	150.0 ± 0.0	92.0 ± 3.8 cd	60.2 ± 7.4 c	29.8 ± 2.5 e
	75 IJs cm^−2^	150.0 ± 0.0	79.8 ± 7.9 ef	47.7 ± 4.5 de	18.8 ± 2.0 f
*H. bacteriophora*	25 IJs cm^−2^	150.0 ± 0.0	120.2 ± 3.9 b	92.9 ± 6.4 b	43.8 ± 4.1 b
	50 IJs cm^−2^	150.0 ± 0.0	98.7 ± 4.5 c	67.3 ± 8.0 c	33.9 ± 2.4 d
	75 IJs cm^−2^	150.0 ± 0.0	86.2 ± 7.1 de	52.1 ± 5.1 d	23.3 ± 1.2 f
d-limonene (60 g/l)	0.5 g/L	150.0 ± 0.0	77.2 ± 4.3 f	44.6 ± 4.3 e	17.1 ± 2.3 f
Control (tap water)	–	150.0 ± 0.0	165.8 ± 4.1 a	172.4 ± 3.5 a	176.8 ± 3.4 a

In each column, averaged means followed by the same letters are not significantly different according to Tukey’s LSD test at α = 0.05.

While young females alive densities in the control plants increased over time, those in plants treated with the above treatments remained very low throughout the experiment (6 DAT: *F*_*index*_ = 605.6, df = 7; 64, P < 0.0001; 12 DAT: *F*_*index*_ = 3899.5, df = 7; 64, P < 0.0001). fewest young female alive was found at 6 and 12 DAT in plants treated with d-limonene at 0.5 g/L, and *S. feltiae* at 75 IJs cm^−2^. The greater reduction rate of young females at 12 DAT was observed in cactus plants treated with d-limonene at 0.5 g/L, and *S. feltiae* at 75 IJs cm^−2^ (*F*_*index*_ = 152.4, df = 6; 56, *P* < 0.0001) (Table 6).

## Discussion

In the current study, the pathogencity of two native EPN isolates collected from Morocco was evaluated against *D. opuntiae* nymphs and adult females, a major threat to cactus production in Morocco under laboratory and field conditions. In laboratory bioassays, the effectiveness of the application of EPNs in the management of *D. opuntiae* was evaluated by considering concentrations and time required for the EPN to kill the host. Our results indicated that *S. feltiae* was the most virulent EPN species against *D. opuntiae*. This EPN at the maximum dose tested (75 IJs cm^−2^) caused the highest mortality of nymphs and adult females after 8 days of exposure which resulted in an LT_50_ value of 5.9 days (nymph) and 6 days (young female), whereas *H. bacteriophora* had the lowest mortalities. Also, a color change in nematode-invaded insects is generally observed for many insect genera^25^, in this study, the scale pests turn dark brown when infected by the two EPN species tested.

These results corroborate those of a previous study by Gorgadze et al.^49^ in which it was reported that the pathogenicity and virulence of an introduced species of Steinernema and *H. bacteriophora* induced mortality rates of 73% in nymphs and 56.5% in adults of brown marmorated stink bug, *Halyomorpha halys* (Stål) (Hemiptera: Pentatomidae). *Steinernema feltiae* and *H. bacteriophora* were also tested together against cabbage weevil, *Rhytidoderes plicatus* Oliv. (Coleoptera: Curculionidae) larvae, and caused 100% mortality in laboratory experiments^50^, being more effective than in the present study. The virulence of the two species of EPN was also evaluated on many other pests including onion thrips *Thrips tabaci* (Lindeman) and tobacco thrips *Frankniella fusca* (Thysanoptera: Thripidae)^27,41,42^. *Heterorhabditis bacteriophora* was the most virulent against sugarcane spittlebugs, A*eneolamia varia* (Fabricius) (Hemiptera: Cercopidae), causing 76% mortality to the insect^51^. *Heterorhabditis bacteriophora* is reported to be virulent (40–86% mortality) against adults of sycamore lace bug, *Corythucha ciliata* (Say) (Hemiptera: Tingidae)^52^. Higher pathogenicity of *S. feltiae* than *H. bacteriophora* against the olive fruit fly larva, *Bactrocera oleae* (Rossi) (Diptera: Tephritidae), was reported by Sirjani et al.^53^. The strains *S. feltiae*-SF-MOR10, *S. feltiae*-SF-MOR9, and *H. bacteriophora*-HB-MOR7 showed significantly higher infectivity (77–80% mortality) and penetration rates against mediterranean fruit fly *Ceratitis capitata* (Wiedemann) (Diptera: Tephritidae) under laboratory and glasshouse conditions^48^. Greater virulence of *H. bacteriophora* (VS strain) and *S. feltiae* (SN strain) were observed against peach fruit fly *Bactrocera zonata* (Saunders) and oriental fruit fly *B. dorsalis* (Hendel) (Diptera: Tephritidae)^45^. Several studies have reported the sensitivity of several scale pest species to EPN. Guide et al.^54^ demonstrated the virulence of isolates from the genera *Heterorhabditis* and *Steinernema* against the coffee root scale *Dysmicoccus* spp. (Hemiptera: Pseudococcidae). *Heterorhabditis zealandica* and *Steinernema yirgalemense* were found to be the most effective candidates for the control of vine mealybug, *Planococcus ficus* and citrus mealybug, *P. citri* (Hemiptera: Pseudococcidae)^38,55^. These differences in nematode pathogenicity may be due to several factors including the specificity of different isolates for different hosts, their efficiency in reaching the host, penetration ability, and its killing efficacy^56^.

In this trial, both isolates tested were found to be pathogenic to nymphs and young females of *D. opuntiae* and their efficacy increased with an increase in concentration. Rahoo et al.^57^ reported that the control of any selected pest by EPN is related to the nematode inoculum concentration, as higher concentrations increase the chance of infections and, therefore, mortality rates. However, we observed that at concentrations of 25, 50, and 75 IJs cm^−2^ mortality was almost similar at 8 DAT in the *S. feltiae* isolate treatment, suggesting that in this isolate treatment, concentrations above 25 IJs cm^−2^ may cause intraspecific competition among nematodes. Selvan et al.^58^ and Gaugler et al.^59^ have pointed out that a minimum density of IJs can circumvent the immune system of the host, to invade and eventually kill it. On the other hand, very high concentrations of EPNs can induce intraspecific competition between nematodes, which reduces their efficiency as biological control agents^60^.

The persistence of entomopathogens in the environment is an important attribute of biological control programs, and some studies have demonstrated the high persistence of several EPN species under field conditions^54,61^. In the field experiments, d-limonene applied at 0.5 g/L and *S. feltiae* applied at 75 IJs cm^−2^ treatments were effective in reducing the numbers of nymphs and adults of the *D. opuntiae*. The numbers of *D. opuntiae* decreased throughout the experiment in the treated plants. *Steinernema feltiae* applied at 75 IJs cm^−2^ significantly reduced the number of adults and nymphs under field trials to less than 20 after 12 DAT. There were also small but significant reductions of *D. opuntiae* adults and nymphs in *S. feltiae* applied at 25 and 50 IJs cm^−2^, and *H. bacteriophora* applied at 75 IJs cm^−2^ (88.6–81.4%) at 12 DAT. Due to low persistent and slow action, *H. bacteriophora* must be applied repeatedly when it is used at low concentrations (authors, personal observations).

Differences in the *D. opuntiae* mortality rates among the EPN species tested could be attributed to the foraging strategy of IJs, as well as the behavior of *D. opuntiae*. *Heterorhabditis bacteriophora* have a cruising strategy^30^ while *S. feltiae* exhibits an intermediate foraging strategy^62^. Also, Bastidas et al.^63^, reported that infection by EPN could be restricted in insects less than 5 mm in length. Studies that used small hemipteran species such as *Pseudococcus viburni* (Signoret) (Hemiptera: Pseudococcidae) and woolly aphid, *Eriosoma lanigerum* (Hausmann) (Hemiptera: Aphididae) reported that the largest stages (1.9–3.0 mm) were more susceptible to EPNs with 38% and 78% mortality, respectively than the smallest stages (0.6–1.2 mm) with 0% and 22% mortality, respectively^64,65^. But for silverleaf whitefly, *Bemisia tabaci* (Gennadius) (Hemiptera: Aleyrodidae), this trend was not observed^66^. These last authors reported second instar nymph mortality rates ranging from 75 to 90%, being the most sensitive life stage than the adult stage for both *Steinernema carpocapsae* (Weiser) (Rhabditida: Steinernematidae) and *S. feltiae* despite its small size (0.8 mm). In our study, *S. feltiae*, at the highest dose, caused 98.8% and 97.5% mortality of nymphs and breeding young females of *D. opuntiae*, respectively. Therefore, the small size of these nymphs (0.70–2.25 mm) and adult females (1.98–3 mm) is not a significant limiting factor for their susceptibility to *S. feltiae*.

For both, nymphs and adult females of *D. opuntiae*, no significant difference was observed among *S. feltiae* at 25, 50, and 75 IJs cm^−2^ and the botanical insecticide, d-limonene applied at 0.5 g/L which was used as a positive control, due to their known toxicity against different stages of *D. opuntiae* and which had high mortality for both nymphs and adult females of *D. opuntiae*^21^, indicating the acceptability of *S. feltiae* as a potential biological control agent against *D. opuntiae*. No previous research has yet been conducted on the control of *D. opuntiae* with EPNs. The compatibility of the use of EPNs and botanical insecticides such as d-limonene in an integrated pest management program against *D. opuntiae* merits to be investigated. In this sense, three agrochemicals and two biocontrol product formulations were found to be compatible with *Heterorhabditis zealandica* and tend to be used in citrus IPM programs^67^. Further studies on the use of adjuvants to improve control with EPN should be performed. Van Niekerk and Malan^68^ showed that the addition of adjuvants prevents nematode desiccation, as well as promotes application deposits on the leaf surface and that merit further evaluation in protect-and-kill strategies in fields that could complement other IPM strategies to improve the management of *D. opuntiae.* Many other biological factors, only some of which were discussed in this study, may affect the final selection of a nematode species or isolate for control of *D. opuntiae* under field conditions. In addition, several environmental factors such as temperature may affect the establishment and pathogenic potential of nematodes under field conditions. Therefore, additional studies using other nematode species and isolates under laboratory and field conditions are needed.

## Material and methods

### The cochineal rearing

The *D. opuntia* was collected from a colony housed at the Entomology Laboratory of the National Institute of Agricultural Research (INRA-Morocco) and used in the trials. *Dactylopius opuntia* were reared according to Aldama-Aguilera and LlanderalCazares method^69^ in cladodes of *O. ficus-indica* collected from the fields in Zemamra, Morocco (32°37′48″ N, 8°42′0″ W) to obtain enough numbers. Each cladode was staked at the basal end with a wooden stake, left to heal for 48 h under laboratory conditions, and then suspended vertically from metal grids in entomological cages (80 cm^3^) consisting of a metal frame covered with mesh fabric to allow ventilation. *Dactylopius opuntiae* gravid females (n = 10) were isolated from infested cladodes and placed in an open wax paper bag (8 cm^2^). Each bag was then attached to the top of each cladode, the remaining cladodes were placed horizontally to support nymphs that were not initially on the vertical cladodes. Infested cladodes were kept in entomologic`al cages (80 cm^3^) under controlled conditions at 26 ± 2 °C, 60 ± 10% relative humidity (RH), and a photoperiod of 12:12 h (Light:Dark) regime.

### Source of Entomopathogenic nematodes

Two EPN species, *Steinernema feltiae* (Filipjev) (Rhabditida: Steinernematidae) (SF-MOR10 strain) and *Heterorhabditis bacteriophora* (Poinar) (Rhabditida: Heterorhabditidae) (HB-MOR8 strain) recently isolated from soil in Morocco (for more information see also Mokrini et al.^48^) were evaluated against *D. opuntia*. EPN isolates were reared at 25 °C according to the methodology described by Kaya and Stock^70^, using the last instar larvae of greater wax moth, *Galleria mellonella* Linnaeus (Lepidoptera: Pyralidae). Dead larvae of *G. mellonella* were placed on white traps^71,72^. Harvested infective juveniles (IJs) were maintained in tap water in tissue culture flask at 8 °C and used within 12 days after harvest.

### Laboratory trials

The pathogenicity of both EPN strains was investigated against *D. opuntiae* nymphs and adult females under laboratory conditions. The trials were carried out in plastic Petri dishes (14.5 cm diameter) (Globalroll) lined with a circular filter paper disc (smooth Sartorius™ quality 3-HW). Ten *D. opuntiae* nymphs (Trial 1) and ten adult females (Trial 2) were transferred to each Petri dish. In both trials, one ml of each EPN species was applied via pipette at the rates of 25, 50, and 75 IJs cm^−2^ (4126, 8252, and 12,387 dish^−1^ respectively). Petri dishes were arranged in a completely randomized design (CRD) with 4 replications. The control Petri dishes received tap water only without the addition of EPNs. Limocide (60 g d-limonene per L; applied at 0.5 g/L; Vivagro, Martillac, France) diluted in tap water was used as a positive control. This botanical insecticide was served as a positive control treatment because of its known toxicity against different stages of *D. opuntiae*^21^. d-limonene (60 g/L), had high mortality against both nymphs and adult females (90.28% and 91.94% mortality, 120 h after treatment respectively) of *D. opuntiae* under field conditions. In addition, the botanical insecticide dose used in the present study was sublethal, as it did not cause short-term mortality to the potential predator of *D. opuntiae*, *Cryptolaemus montrouzieri* (Mulsant) (Coccinellidae: Scymninae)^21^. Numbers of alive and dead scale insects were recorded at 1, 2, 4, 6, and 8 days after application. The dead insects were observed under binocular loupe (SFC-11, MOTIC^®^) for the presence EPNs inside the cadavers. To ensure the reproducibility of results, all experiments were independently repeated twice over time (two full trials with 8 replicates total).

All experiments were conducted under similar conditions of 26 ± 2 °C, 60 ± 10% RH, and a photoperiod of 12:12 h (Light:Dark) at room temperature. The mean body weights and sizes of *D. opuntiae* nymphs and adults used in the studies were 3.8 ± 0.5 mg and 0.7–1.6 mm, respectively (nymphs) and 5.2 ± 0.2 mg and 1.98–2.25 mm, respectively (young adults females).

### Field trials

Field trial was carried out in an *O. ficus-indica* area in the experimental field station (32°15′ to 33°15′ N, 7°55′ to 9°15′ W) at the INRA Settat (National Institute of Agricultural Research), Morocco, during the 2020–2021 growing season. This trial was conducted on a half-hectare plot planted with 200 cladodes (1-year-old) of *O. ficus-indica,* susceptible to *D. opuntiae*. The collected cladodes of *O. ficus-indica* used in the experiments were conducted in accordance with the guidelines and regulations of the Moroccan Agriculture Ministry. The cladodes were planted in normal polarity in completely randomized rows (1 m between rows, with a spacing of 0.5 m between plants), and were grown until they reached the stage of three to five cladodes. The plot had a total of 15 rows and each row had 13 plants. The plants were irrigated as needed. It should be noted that this experiment was installed in an environment of cactus hedges completely infested or even devastated by the cochineal *D. opuntiae*.

The plants were infested with 1-day-old first instar nymphs of *D. opuntiae* that were allowed to settle in before being adjusted to appropriate densities. To standardize treatments and replicates, only 150 nymphs and 150 young females identified with the help of a hand-held magnifying glass were retained on the plants; additional nymphs and adults were removed using a needle^21^.

The same EPN strains tested in the laboratory bioassays at different concentrations were evaluated in the field experiment using the same concentrations (25, 50, and 75 IJs cm^−2^). There were eight treatments (five plants were treated by each treatment). The EPNs and d-limonene solutions were applied using a laboratory sprayer (Burkard Scientific Ltd, Uxbridge, UK) to ensure complete coverage. Nematodes were applied in 500 ml of tap water in a 0.5 m^2^ area around the base of each plant. Plants were examined with a hand-held magnifying glass, 1 day before treatment, and 3, 6, and 12 days after treatment (DAT), and the number of alive *D. opuntiae* was counted. Five plants per treatment were considered as a replicate, and four replicates were conducted for all treatments, arranged in a randomized complete block design (RCBD). This trial was repeated twice over different time.

The rate of population reduction at each sampling date was calculated by the Henderson-Tilton formula^73^:

$$R({\%})=\left[1-\frac{T2 \times C1}{T1 \times C2}\right] \times 100,$$

where T1 and T2 are respectively the numbers of insects alive on the treated plant before treatment and on a specific sampling date after treatment, while C1 and C2 are the numbers of insects alive in a control plant before treatment and on a specific sampling date after treatment, respectively.

### Statistical analysis

The mortality percentage data for each treatment in the laboratory bioassays were corrected using the Abbott formula^74^. The corrected mortality percentage data were subjected to ANOVA and means were separated using Tukey's LSD test (α = 0.05).

The probit analysis method was established to determine the lethal concentration (LC_50_) for the different treatments using IBM SPSS 23.0 software. Mortality data were transformed into probits, while concentrations were transformed into Probit log10 (dose). Before analysis, LC_50_ values were predicted from the probit lines. The method of Finney^75^ was used to determine the lethal time (LT_50_) of the probit analysis. Calculation of the lethal concentration (LC) and its 95% confidence limits (CL) was performed based on accurate estimation of log (CL) variances^76^. The Kaplan–Meier survival analysis technique was used to describe both, the median lethal time (LT_50_) (the number of days until 50% of the insects were dead, for each treatment) and the mean survival time (SPSS 23.0).

One-way ANOVA test was performed to examine differences between doses and exposure times using the SPSS 23.0 package at the levels of P < 0.05 and P < 0.01. Significant differences between variables were checked using Tukey's LSD test.

The numbers of adults and nymphs alive, and rates of population reduction in the different treatments in the field experiment were subjected to ANOVA under RCBD, and means were separated by Tukey's LSD test. All tests were performed using SPSS 23.0 software^77^. In all experiments, treatment was considered a fixed effect, and replicate was considered a random factor.
